# Endothelial cell leptin receptors, leptin and interleukin-8 in the pathogenesis of preeclampsia: An in-vitro study

**DOI:** 10.4274/tjod.78545

**Published:** 2017-12-30

**Authors:** Sefa Arlıer

**Affiliations:** 1 South Florida University Faculty of Medicine, Department of Obstetrics and Gynecology, Tampa, USA; 2 University of Health Sciences, Adana Numune Training and Research Hospital, Clinic of Obstetrics and Gynecology, Adana, Turkey

**Keywords:** Preeclampsia, endothelial cell, umbilical cord, leptin, leptin receptor, interleukin-8

## Abstract

**Objective:**

Increased leptin hormone and leptin receptor may enhance the generation of proinflammatory cytokines by endothelial cells and lead to endothelial dysfunction. This study assessed the umbilical cord endothelial leptin receptor levels in preeclampsia and investigated the effect of leptin on endothelial interleukin-8 (IL-8) production.

**Materials and Methods:**

The association between IL-8 levels with leptin stimulation was investigated in leptin-treated human endothelial cells. Endothelial cell leptin receptor levels were evaluated using immunohistochemistry staining, and endothelial IL-8 protein expression by Western blot analysis. Data are presented as mean ± standard error of the mean (SEM). Statistical significance was analyzed using Student’s t-test or Mann-Whitney U test and one-way analysis of variance.

**Results:**

Leptin receptor immunoreactivity increased significantly in umbilical cord venous and arterial endothelial cells in normal pregnancy (n=12) compared with preeclampsia (n=7) endothelial cells. The corresponding preeclampsia versus control histologic scores (mean ± SEM) were 67.9±8.8 vs. 127.6±23.1, (p=0.011) for the leptin receptor and 55.4±8,0 vs. 93.7±17.1 (p=0.035), respectively, for the vein endothelial cells. Leptin treatment significantly increased IL-8 protein levels (control vs. 100 and 1000 ng/mL, p=0.003).

**Conclusion:**

The findings of increased umbilical cord endothelial leptin receptor levels in preeclampsia and increased endothelial IL-8 expression with exposure to higher leptin concentrations may indicate the contribution of leptin to endothelial dysfunction and increased neutrophil-endothelial interaction, which are significant pathophysiologic features of preeclampsia.

## PRECIS:

This study identifies, higher levels of leptin expressions in umbilical cord from preeclampsia placenta, suggesting that provocation of interleukin-8 secretion by leptin in endothelial cells may contribute to preeclampsia-related inflammation.

## INTRODUCTION

Preeclampsia (PE) is a multisystem disorder. Hypertension, proteinuria, and pathologic edema in pregnancy are the classic clinical manifestations. Worldwide, approximately 5-7% of primigravid women develop PE in pregnancy^(1)^. Many studies implied that the fundamental pathophysiologic abnormality is endothelial dysfunction associated with exaggerated inflammation and immunologic reaction^(2,3,4)^. In the pathogenesis of PE, activation of neutrophils, monocytes, and natural killer cells initiate inflammation, resulting in endothelial destruction in the pathogenesis of PE^(4)^. The damaged endothelium produces various chemokines, one of which is interleukin (IL)-8^(5)^.

Leptin was initially defined as an adipocyte hormone that controls energy balance, reproductive functions, and immune reactions in the body^(6)^. Mounting data imply that leptin is a novel proinflammatory adipocyte-originated element that manages the cytokine pathway by connecting the immune and inflammatory system^(7,8)^. The plasma leptin concentrations in patients with PE are considerably higher than those in normal patients^(9)^. Moreover, recent reports revealed that leptin showed vital roles in diverse physiologic processes including angiogenesis and arterial blood pressure regulation^(9)^.

IL-8 has been reported to activate chemotactic migration, proliferation, and survival of vascular endothelial cells; induce the expressions of vascular endothelial growth factor (VEGF) and VEGF receptor^(10)^, and regulate pathologic angiogenesis^(11)^. Was first purified as a potent neutrophil chemotactic factor^(12)^. IL-8 is also characterized as a proinflammatory cytokine. Increased IL-8 levels have been described in various inflammatory diseases^(13)^. Many inflammatory cells, such as monocytes, lymphocytes, and mast cells, release IL-8 that is accumulated inside endothelial cells. IL-8 and its receptors C-X-C chemokine receptor 1-2 have been detected in endothelial cells^(14)^. Moreover, human placental tissue produces IL-8 during pregnancy. Previous studies showed a robust correlation between IL-8 levels and the severity of PE^(5,15)^. Neutrophil activation results in progressive damage to endothelial cells^(16)^. The present study aimed to investigate umbilical cord (UC) leptin receptor (LEPR) levels in PE and to examine the direct effect of leptin on IL-8 production by human endometrial endothelial cells (HEECs) and human umbilical vein endothelial cells (HUVECs), thus to describe the function of leptin in PE.

## MATERIALS AND METHODS

### Tissue collection

Serial paraffin sections of human placental UC specimens were obtained from the University of South Florida under the protocol approved by the Ethics and Human Investigation Committees of the University of South Florida (approvel number: 00015578). Written and verbal informed consents were obtained from each patient. All of the samples were grouped according to clinical diagnosis: UC control (n=12) or PE (n=7). For in vitro studies, previously frozen HEECs (n=2) and HUVEC (n=1) from normal women undergoing hysterectomy (laparoscopy or laparotomy) or normal delivery were thawed and grown to confluence, as previously described^(17)^.

### Immunohistochemistry

Collected PE and normal patient UC paraffin blocks were cut into 5 µm sections that were then put into a heater to incubate overnight at 56 °C. The slides were deparaffinized in xylene (x3) for 20 min., followed by 100, 90, 80, and 70% alcohol x1 for 10 min. per gradient. Following deparaffinization, the slides were heated in 10 mM citrate buffer (pH 6.0) for 3x5 min in a microwave oven for antigen retrieval. The slides were then immersed in 3% hydrogen peroxide (in 1:1 v/v methanol/distilled water) for 12 min. to quench endogenous peroxidase activity. After washing with tris-buffered saline (TBS); (pH: 7.4) (x3) for 5 min., the slides were incubated in a humidified chamber with 5% blocking normal goat serum (Vector Labs, Burlingame, CA) for 30 min at room temperature (RT) in TBS. Excess serum was emptied, and then the slides were incubated overnight with a primary rabbit polyclonal anti-LEPR antibody (1:60; Santa Cruz Biotechnology, Dallas, TX) in 1% normal goat serum at 4 °C. Normal rabbit immunoglobulin G (IgG) (Vector Labs) isotypes were used for negative controls at the equal primary antibody concentrations. The slides were rinsed (x3) for 5 min. with TBS, and then biotinylated anti-rabbit IgG (Vector Labs) was used at a 1:400 dilution for 30 min. at RT. The antigen-antibody complex was identified using an avidin-biotin-peroxidase kit (Vector Labs) for 30 min. at RT. 3.3´-Diaminobenzidine tetrahydrochloride dihydrate (Vector Labs) was added as the chromogen to visualize immunoreactivity for 90 seconds. The slides were then counterstained with hematoxylin and mounted. Immunoreactive LEPR levels were semi-quantitatively assessed using the subsequent intensity categories: 0, no staining; 1+, weak but visible staining; 2+, moderate or distinct staining; and 3+, strong staining. As described previously^(18)^, for each tissue, a histologic score (HSCORE) was derived by adding the percentages of cells that were stained at each intensity category and then multiplying that value by the weighted intensity of the staining using the formula HSCORE=Σ Pi (i + l), where i represents the intensity scores, and Pi is the corresponding percentage of the cells. In each slide, three randomly selected areas were assessed under a light microscope (x40 magnification), and the percentage of the cells at each intensity within these regions was evaluated at different times by two blinded researchers. The average HSCORE of the two examiners was used. Human endometrial endothelial cell and human umbilical vein endothelial cell isolation and experimental treatment with leptin. Frozen primary HEECs (n=2) and HUVECs (n=1) were derived from banked samples. The samples had been isolated and categorized as previously described^(17)^ from endometrial specimens obtained from reproductive-age women undergoing hysterectomy (laparoscopy or laparotomy) and UC vein specimens obtained from the delivery of a normal pregnant woman. Written informed consent for sample retrieval was obtained from Yale University Faculty of Medicine, Human Investigation Committee and approved by the University of South Florida. Aliquots of frozen primary HEECs and HUVECs were thawed and grown to confluence in basal medium (BM), and a phenol red-free 1:1 v/v Dulbecco’s Modified Eagle Medium/Ham’s F-12 (Gibco, Grand Island, NY) mixture, containing 100 U/mL penicillin, 100 μg/mL streptomycin, and 0.25 μg/mL Fungizone complex (Gibco) supplemented with 10% charcoal-stripped calf serum (Gibco). At ~80% confluence, HEECs and HUVECs were transferred to 6-well plates at a density of 150x103 cells/well, for the corresponding treatments, as designated by each experimental condition. Confluent HEECs and HUVECs were incubated in parallel in BM with 0.1% ethanol (vehicle control) or leptin (0.1, 1.0, 10, 100 and 1000 ng/mL leptin, respectively). After incubation for 24 h, HEECs and HUVECs were rinsed with ice-cold 1x phosphate buffered saline and stored at -80 °C until used for immunoblotting analysis to measure IL-8 total protein levels.

### Immunoblot analysis

Total protein was extracted using a cell extraction buffer (BioSource International, Camarillo, CA) containing 3 mM phenylmethylsulfonyl fluoride and a protease inhibitor cocktail (Sigma-Aldrich, St. Louis, MO). The protein level was determined using a detergent-compatible protein assay (Bio-Rad, Hercules, CA). Samples (40 µg) were loaded on 10% Tris-hydrochloric acid-ready gels (Bio-Rad), electrophoretically separated, and electroblotted onto a nitrocellulose membrane (Bio-Rad). The membrane was blocked with 5% non-fat milk powder in TBS containing 0.1% Tween 20 (TBS-T) for 1 h to reduce any non-specific antibody binding. Subsequently, the membrane was incubated overnight with a monoclonal mouse IgG1 clone primary antibody against IL-8 (1:800 R&D Systems, Inc., Minneapolis, MN) in 5% non-fat milk powder in TBS-T. The membrane was then rinsed several times with 1x TBS-T for 1 h and incubated with horseradish peroxidase-conjugated anti-rabbit IgG (Vector Labs) in TBS-T. Following several washes, IL-8 was visualized through light emission from the film (Denville Scientific, Holliston, MA) with enhanced chemiluminescence substrate (Thermo Scientific, Rockford, IL). Band intensities were quantified using computer densitometry analysis (Image J, National Institutes of Health, Bethesda, MD).

### Statistical Analysis

Data from immunohistochemistry and Western blot analysis that were normally distributed, according to the Kolmogorov-Smirnov test, were compared using Student’s t-test or one-way analysis of variance, followed by the post hoc Holm-Sidak test. Immunohistochemistry and Western blot analysis data that were not normally distributed were analyzed using the Kruskal-Wallis nonparametric ANOVA-by-Ranks test, followed by the post hoc Student-Newman-Keuls test. Statistical calculations were performed using Sigmaplot 13 for Windows (Jandel Scientific Corp., San Rafael, CA). Statistical significance was considered as p<0.05.

## RESULTS

### Immunohistochemistry of human placental umbilical cord specimens evaluating leptin receptor immunostaining in the control vs. preeclamptic groups

**Figure 1:**
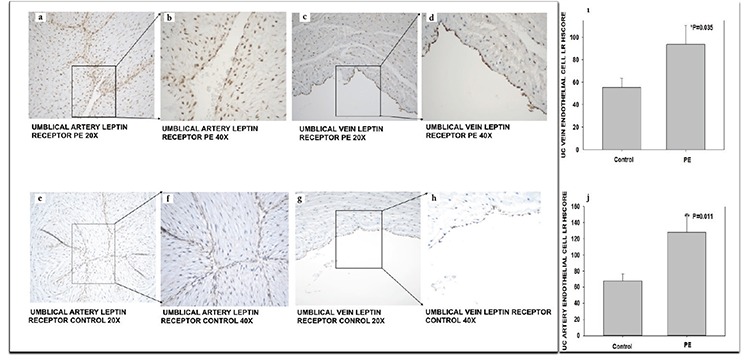
Leptin receptor immunoreactivity in umbilical cord arterial and venous sections from gestational age-matched preeclamptic and normal pregnancies. Representative micrographs of immunohistochemical staining for leptin receptor in preeclampsia (n=7) (A, B, C, D) and normal pregnancy (n=12) (E, F, G, H). Graphs represent the histologic score analysis of leptin receptor immunostaining in umbilical cord vein (I) and umbilical cord artery (J) endothelial cells expressed as mean ± standard error of the mean *p=0.035; endothelial cells of the umbilical vein, preeclampsia vs. normal pregnancy **p=0.011; endothelial cells of the umbilical artery, preeclampsia vs. normal pregnancy PE: Preeclampsia

LEPR immunostaining was detected in the cytoplasm and nucleus of the UC artery and vein endothelial cells. LEPR HSCOREs were significantly different between the control vs. PE specimens in the sectioned UC arterial endothelial cells [mean ± standard error of the mean (SEM): 67.9±8.868 vs. 127.6±23.1; p=0.011, respectively] (Figure 1) and UC vein endothelial cells (mean ± SEM: 55.4±8.043 vs. 93.7±17.15; p=0.035, respectively). LEPR immunostaining was moderate in the PE specimens (n=7) (Figure 1a-d), whereas the control UC endothelial cells (n=12) displayed weak immunostaining (Figure 1e-h). There were no statistically significant differences in maternal age, gestational age (GA), body mass index (BMI) at delivery, and baby weight between the control and PE groups (Table 1).

### Effect of leptin on interleukin-8 protein expression in cultured human endometrial endothelial cells and human umbilical vein endothelial cells

**Figure 2:**
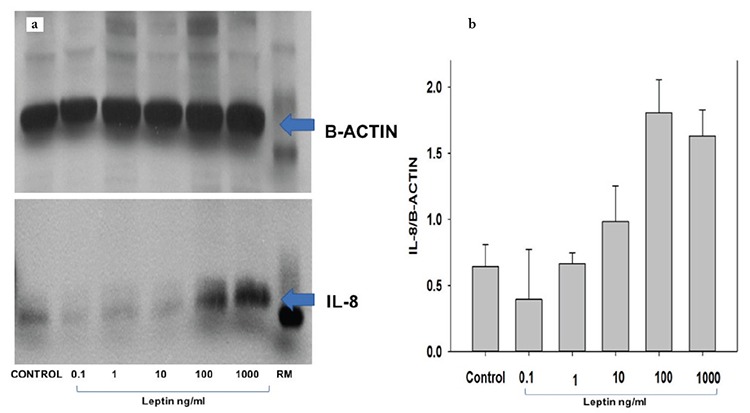
Leptin stimulates interleukin-8 expression in endothelial cells. Representative immunoblotting in human endometrial endothelial cells (n=2) and human umbilical vein endothelial cells (n=1) cultures treated with leptin for 24 hours. Western blot analysis demonstrating the effect of leptin on interleukin-8 levels in human endometrial endothelial cells and human umbilical vein endothelial cells. Confluent human endometrial endothelial cells and human umbilical vein endothelial cells cultures were treated with vehicle (control), 0.1 1, 10, 100 and l000 ng/mL leptin, respectively, for 24 hours, to evaluate the effect of leptin. Immunoblot bands for interleukin-8 were quantified using Image J. Bars represent mean ± standard error of the mean (n=3) *p<0.05; both for 100 and 1000 ng/mL leptin vs. control. Data are representative of three independent experiments IL: Interleukin

Experimental incubations were followed by immunoblotting of the cell extracts to establish the functional regulation of leptin on IL-8 protein expression in the primary cultures of HEECs and HUVECs. Representative immunoblotting (Figure 2a) and the accompanying graphs (Figure 2b) indicated that IL-8 protein level was significantly increased by 1000 ng/mL leptin and 100 ng/mL compared with the control (p=0.003). Conversely, 0.1, 1, and 10.0 ng/mL leptin had not statistically significant effect on IL-8 levels when added to the culture medium (p>0.05) (Figure 2a,b). Incubation with 100 and 1000 ng/mL leptin induced greater IL-8 protein level vs. the control, 0.1 and 1 ng/mL leptin (Figure 2a). Compared with the control, 0.1, 1, and 10 ng/mL leptin showed no significant change in the basal IL-8 protein expression in HUVECs and HEECs.

## DISCUSSION

Several studies illustrated the potential role of cytokines, chemokines, and their receptors in the development and progression of PE^(19,20,21)^. Pregnancy complications associated with PE are major causes of materno-fetal morbidity and mortality, but their pathogenesis remains unclear^(22)^. Despite significant development in the management of PE, it still constitutes an unsolved health problem in pregnancy^(23)^. The current study examined the molecular mechanism of leptin-mediated inflammation in PE. The data in this study imply that leptin/LEPR interaction stimulated IL-8 production in endothelial cells, consequently promoting neutrophil chemotaxis and PE progression. Therefore, a view was instigated to connect leptin concentrations and IL-8 level in endothelial cells. The structure and function of endothelial cells are crucial to the preservation of arterial and vein vessel wall homeostasis, as well as immune cells migration^(24)^. Damaged endothelial cells, increasing inflammatory cell recruitment, and high concentrations of inflammatory agents in the plasma of pregnant patients are responsible for the pathogenesis of PE^(13,24)^. Impaired placental function and placental vascular disorders result in the occurrence of poor perinatal outcome^(25)^. Leptin is produced by cytotrophoblasts and syncytiotrophoblasts in the human placenta and adipose tissue, which is then secreted into the circulation, where it exerts its effects via interaction with the LEPR. The effect of leptin on endothelial cells has attracted particular attention^(20,26)^. LEPRs are expressed in many normal tissues, but also in pathologic tissues generally associated with obesity and abnormal energy balance^(27)^. Immunohistochemically, endothelial cells, syncytiotrophoblasts, and cytotrophoblasts were stained with leptin^(28)^. The present study demonstrated that the LEPR expression in UC artery and vein endothelial cells was augmented in PE and localized to the cytoplasm and nuclei of endothelial cells. Significant increases in the concentration and amounts of LEPR-positive endothelial cells, as implied by HSCORES in PE vs. normal pregnancy suggests a function for LEPR in the inflammatory pathway of PE. A previous study demonstrated a high level of the leptin gene expression in microarray investigations in PE^(29)^. In this regard, the current study was designed to elucidate the possible correlation between UC artery and vein LEPR levels and PE and its functional effects on endothelial cells. The study data can also be considered as indirect evidence for LEPR and its contribution to PE, and the functional results of leptin on endothelial cells. The data of the study can also be considered as indirect evidence for the contribution of LEPR to PE. Leptin possibly has an effect on the regulation of arterial blood pressure in pregnant women, as indicated by the direct relationship between plasma leptin concentrations and mean arterial blood pressure^(30)^. Furthermore, dysfunctions of leptin metabolism or regulation in the placental unit and plasma are enhanced in pregnancies complicated with various abnormalities such as intrauterine growth restriction, gestational diabetes mellitus, and PE. Leptin synthesis and secretion have been shown as positively correlated with BMI and GA^(31,32,33)^. In contrast, in this study, BMI, GA, maternal age, and birth weight of the baby were not significantly different between the control and PE groups. Mise et al.^(34)^ stated that leptin messenger RNA (mRNA) levels in severe PE were significantly higher than in those with mild PE, also that placental leptin mRNA levels were generally similar to plasma leptin concentrations in women with PE compared with GA-matched healthy pregnant women. Higher concentrations of UC plasma leptin have been revealed in infants of mothers with PE than in a GA-, sex-, and infant ponderal index-matched control group^(35)^. Compromised placental blood distribution causes chronic disturbance of nutrient resource and ultimately results in intrauterine growth restriction^(36)^. Impaired placental perfusion also creates a depressed oxygen source at the placental level, which subsequently enhances leptin gene expression in the placental unit^(21)^. It is likely that the high leptin concentrations in maternal plasma may augment hypertension because leptin provokes endothelial dysfunction and hypertension via aldosterone-related mechanisms and milieu in gestations complicated by intrauterine growth restriction^(37)^. PE is concomitant with shallow trophoblastic invasion into the endometrial layer, which leads to poor placental perfusion and augmented fetal and maternal plasma leptin levels that are considerably increased over the concentration of leptin specific to human gestation^(38)^. This exaggerated hyperleptinemia may be linked to a compensatory response to augment nutrient supply to the growing fetus^(39)^. The present study showed that although not significant, the LEPR level was slightly higher in the UC artery than the paired UC vein, especially in the preeclamptic group, suggesting that UC artery LEPR may be more functional than UC vein LEPR in PE. In a previous report, leptin levels were found to be higher in the UC vein than the UC artery^(40)^. One other study showed that the leptin concentrations were considerably higher in UC artery and UC vein than those in paired maternal plasma, implying that leptin is produced in placental trophoblastic cells and is secreted into the maternal blood circulation. Furthermore, plasma leptin concentrations in the UC vein were notably higher than those in the paired UC artery, suggesting that leptin is released from placental trophoblastic cells into the fetal blood circulation^(41)^.

The increase in the proinflammatory chemoattractant cytokine levels in PE suggests an inflammatory basis for this disease. The proinflammatory and regulatory cytokine IL-8 has been discovered in endothelial cells. Prior studies demonstrated upregulation of IL-8 protein levels in PE. IL-8 is regulated by neutrophil and monocyte chemotaxis regarding the inflammation site and stimulated inflammatory response^(42)^. IL-8 is secreted by some cell types, including endothelial cells, monocytes, macrophages. Neutrophils, and fibroblasts. This study investigated UC LEPR levels in PE and the relationship of IL-8 expression in HEECs and HUVECs with leptin at different concentrations in an attempt to explain the significant association between leptin and IL-8 in patients with PE. Increasing placental endothelial IL-8 production may contribute to the improvement of placental endothelial pathology in PE. There was a dose-dependent progressive connection between increasing leptin level and endothelial IL-8 protein expression. Exposing endothelial cells to a high leptin concentration plays a role in leukocyte migration into the placental area and in the management of the tissue-specific modifications related with the leukocyte extravasation. A prior study showed that circulating plasma IL-8 concentrations were elevated in women with PE compared with normal pregnant women^(5,15)^. These data indicate that IL-8 may have a critical role in endothelial cell proliferation and differentiation and regulating endothelial function. Human placental tissues constitutively produce IL-8 in pregnancy, and IL-8 secretion increases with progressing GA. IL-8 is critical for leukocyte recruitment^(43)^. Namely, the vascular endothelial cell layer acts as the gatekeeper for maternal immune rejection and immune cells. Similarly, increased IL-8 plasma levels in PE have been documented ^(15,44)^. Moreover, leptin induced the production of IL-8 in human cartilage, fibroblasts, and M2 macrophages^(45,46)^. These previous data also support the role of leptin-induced IL-8 secretion in endothelial cells^(46)^. Additionally, the current study is the first to describe the association between leptin and IL-8 in HUVECs and HEECs. IL-8 influences early vascular remodeling by recruiting circulating neutrophil cells to the endothelial cells^(24)^. Understanding the mechanism for the increased IL-8 expression in HEECs and HUVECs of women in pregnancy may contribute to explaining the pathophysiology and development of PE. Speculatively, increased leptin production may exaggerate cytokine-induced destruction of endothelial cells in PE or overweight patients. Neutrophil-endothelial contact is a hallmark of vascular inflammation that results in endothelial injury/dysfunction^(47)^. Increasing IL-8 levels in endothelial cells probably contribute to enhanced neutrophil recruitment and cytokine production. Furthermore, neutrophil stimulation is amplified in inflammatory reactions in the maternal artery and vein blood circulation in PE. These observations reveal that there is a high rate of IL-8 production in PE, consistent with other studies^(44)^. However, further studies are required to investigate the role of leptin and LEPR in other inflammatory-related cellular mechanisms.

Collectively, these in vivo and in vitro results indicate that endothelial cells contribute to increased IL-8 concentrations in maternal and fetal circulation, as well as neutrophil recruitment. Leptin-associated augmented IL-8 secretion in endothelial cells is probably related with the development of PE. Thus, the increase in IL-8 level may potentiate leukocyte activation into the placental tissue under the effects of leptin. Consequently, the source and physiologic importance of IL-8 in the maternal and fetal circulation are noteworthy objectives of potential investigation. However, whether this phenomenon is a compensatory effect or amplified reaction to the severity of the PE remains enigmatic^(48)^.

## CONCLUSION

These observations provide direct evidence of the stimulation of IL-8 gene expression in endothelial cells in vitro by high leptin concentrations. In this manner, the increased leptin and LEPR may cause or contribute to increased IL-8 production, leading to increased neutrophil recruitment and endothelial destruction and consequently, increase cytokine expression in PE. This study may provide an in vivo basis for the application of an anti-human IL-8 antibody for the treatment of PE. Further studies researching the possible role of LEPR in normal and PE UC endothelial cells are needed to explore these possibilities and to support new insight into our understanding of the pathogenesis of PE.

## Figures and Tables

**Table 1:**
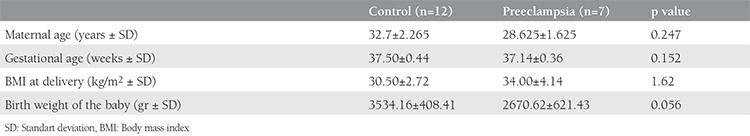
Demographic data of the control and preeclamptic groups from which umbilical cord specimens were obtained
